# Assessing a computational pipeline to identify binding motifs to the *α*2*β*1 integrin

**DOI:** 10.3389/fchem.2023.1107400

**Published:** 2023-02-13

**Authors:** Qianchen Liu, Alberto Perez

**Affiliations:** Department of Chemistry and Quantum Theory Project, University of Florida, Gainesville, FL, United States

**Keywords:** molecular recognition, integrin, AlphaFold, moecular modeling, binding

## Abstract

Integrins in the cell surface interact with functional motifs found in the extracellular matrix (ECM) that queue the cell for biological actions such as migration, adhesion, or growth. Multiple fibrous proteins such as collagen or fibronectin compose the ECM. The field of biomechanical engineering often deals with the design of biomaterials compatible with the ECM that will trigger cellular response (e.g., in tissue regeneration). However, there are a relative few number of known integrin binding motifs compared to all the possible peptide epitope sequences available. Computational tools could help identify novel motifs, but have been limited by the challenges in modeling the binding to integrin domains. We revisit a series of traditional and novel computational tools to assess their performance in identifying novel binding motifs for the I-domain of the *α*2*β*1 integrin.

## 1 Introduction

**FIGURE 1:**
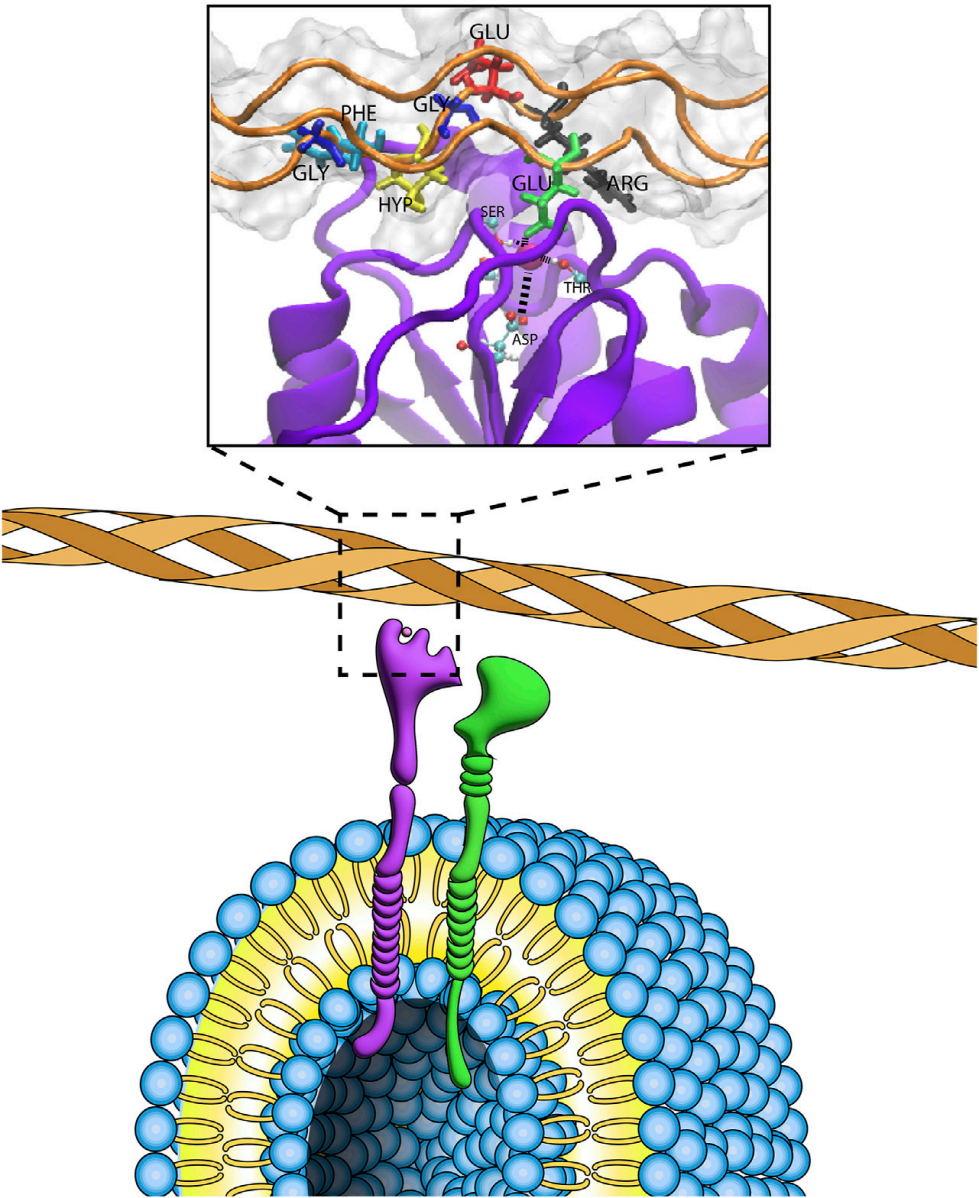
Sytem of study. Artistic representation of the *α*2*β*1 binding collagen. The inset corresponds to the PDB structure 1dzi, focusing on the specific motif area “GFOGER” on the collagen fiber (orange).

The integrin superfamily (Hynes, 1987) encompasses 24 different integrins in humans responsible for communication and singaling between cells and with the extracellular matrix (ECM). Structurally, they are *αβ* heterodimers with two non-covalent subunits (arising from 18 *α* and 8 *β* subunits) located on the cell’s membrane (Hynes, 2002; Takada et al., 2007). Their normal behavior controls cellullar processes such as cell adhesion, migration and differentiation [(Critchley et al., 1999); (Mizuno et al., 2000); (Mercurio et al., 2001)]. Usually, these integrins recognize specific peptide epitope motifs present in large fibrous proteins that form the extracellular matrix such as collagen or fibronectins (see Figure 1). Hence, designing molecules that disrupt or enhance these interactions has long been a potential therapeutic target. A recent study (Slack et al., 2022) shows over 60 integrin-target therapies have been recorded (https://www.clinical-trials.gov and https://www.clinical-trialsregister.eu/ctrsearch/search using the search term “integrin”) targeting diseases like Multiple Sclerosis (Kawamoto et al., 2012) or Crohn’s disease (Hutchinson, 2007). Most binding occurs through an “I-like domain” in the *β* subunit which contains a “metal ion-dependent adhesion site” (MIDAS). Some peptide epitope binding motifs like RGD (Arginine-Glycine-Aspartic) are present in many ECM fibers and bind many integrins (Hatley et al., 2018). However, there is selectivity and specificity among their ligands—and even for the RGD motif there is an interplay between the conformation it adopts and the specificity to a particular integrin (Aumailley et al., 1991; Kapp et al., 2017). In the field of biomaterial engineering, there is growing interest to develop computational pipelines that can identify functional motifs to incorporate into engineered ECMs that trigger cellular response (Perez et al., 2021).

Our existing understanding of integrin-ligand recognition has mainly been driven from experimental observations including affinity chromatography Otey et al. (1993), antibodies against cell epitopes Ley et al. (2016), and the use of NMR experiments Siebert et al. (2010). Computational tools on the other hand have been challenged by the complexity of modeling integrin-ECM interactions as well as the diversity of function/structure relationships arising from the multidomain architecture that merit attention such as the origin of selectivity, mechanism for signal transduction (Kalli et al., 2011), effect of the lipid environment (Kalli et al., 2017), or interaction between the different domains and their role in active/inactive conformations to name a few (Chen et al., 2011). Although the number of computational studies for integrin systems is limited, there is a wide range of approaches that have been used including physics based approaches such as docking (Guzzetti et al., 2017), atomistic and coarse grained molecular dynamics (MD) (Craig et al., 2004; Murcia et al., 2008; Choi et al., 2010; Zhu et al., 2010; Wang et al., 2015; Farina et al., 2016; Fratev and Sirimulla, 2019), QM/MM approaches (Freindorf et al., 2012), and machine learning (Mehdi et al., 2013; Prytuliak et al., 2017; Asgari et al., 2019). Typically, ligand docking calculations are applied to filter ligands with high affinity, MD approaches are used to either predict free energy differences with thermodynamic integration (TI) or conformational changes *via* enhanced sampling, while ML approaches have been traditionally used to discover new binding motifs in protein-peptide complexes such as the well-known RGD, GPR (the recognition site for *α*x*β*2), or DLLEL (the binding site for *α*v*β*6) for integrins.

We focus on the I-domain of the *α*2*β*1 integrin, which contains a binding motif and has been shown to retain the binding activity of the whole integrin in recombinant studies expressing only the I motif (PDB code 1dzi) (Emsley et al., 2000). The binding domain undergoes a conformational change between the unbound and bound forms in which three loops participate in coordinating a central metal ion, with a glutamic acid from the collagen completing the coordination of the metal (Emsley et al., 2000). The collagen used here introduces a six aminoacid peptide motif (*GFOGER*, where O stands for hydroxyproline), that forms triple helices analogous to canonical collagen. Even though the three strands are homologous for triple-helix formation, during binding each strand becomes distinct, with one containing a critical Glutamic acid residue (E) for binding (“leading strand”). By comparison, the other two strands have been previously named “middle” and “trailing” strands) (Emsley et al., 2000). Given the 20^6^ possible peptide sequences covering the length of the *GFOGER* motif, we expect there are many other sequences that might bind this integrin. Indeed, amongst integrins that bind collagen, there are differences amongst canonical motifs (*GxOGER*, where x = F, L, M, A) and non-canonical motifs (Hamaia et al., 2012). Hence we ask the question of whether computational pipelines can suggest new motifs and if they are capable of assessing which of those suggested motifs are actually better binders.

We seek to assess the advantages/disadvantages of using traditional and novel pipelines combining multiple computational techniques readily available. We divide the pipelines in three stages: 1) predicting new motifs, 2) predicting their ability to bind, and 3) predicting their stability. Overall, finding new interacting motifs against integrins remains challenging regardless of the pipeline used.

## 2 Computational methods

### 2.1 Identification of novel motifs

We started from the X-ray crystal structure of the *α*2 I domain from *α*2*β*1 in complex with collagen [PDBid: 1dzi (Emsley et al., 2000)] and performed a scan of all possible mutations (for the 20 common amino acids) at each position along the “GFOGER” motif, collecting the expected free energy changes (ΔΔG) these programs predict. Integrin complexes were first optimized in the FoldX suite. Next, a position scan was conducted with the command “Position Scan” on the *GFOGER* motif and the output results showed the difference of binding energy for each mutation per amino acid on collagen. ΔΔ*G*_*bind*_ was also calculated using *RosettaDDG* predictions, with the backrub trajectory stride set to 35,000 and making three trials for each ΔΔ*G* calculation.

The ProteinMPNN (message passing neural network) (Dauparas et al., 2022) has recently been developed as a way to identify the ideal sequence that will adopt a certain 3D structure. In this model, we provided the PDB structure of the complex and asked the model to design new motifs to replace the native *GFOGER* motif.

### 2.2 Stability MD simulations

We used standard minimization and equilibration protocols (Braun et al., 2018) followed by production runs using Langevin dynamics for 500 ns in the NPT ensemble using a Monte Carlo barostat (Åqvist et al., 2004). Simulations used AMBER’s (Case et al., 2020) *pmemd* module (Salomon-Ferrer et al., 2013). We simulated the top 20 FoldX and Rosetta predictions using ff14SB (Maier et al., 2015) solvated in a truncated octahedron box [OPC water model (Izadi et al., 2014)], and 150 mM concentration of Na^+^ and Cl^−^ ions (Joung and Cheatham, 2008). As a control, we simulated the I-domain in the presence and absence of the wild type (WT) collagen (PDBid: 1dzi). All simulations were carried out with a Co^2+^ ion in the MIDAS binding site. We simulated 10,000 steps of energy minimization, switching from steepest descent to conjugate gradient after 5,000 cycles. The resulting minimized system was heated from 0 to 100 K in NVT condition for 50 ps with Langevin dynamics, and 100–300 K in NPT for 500 ps using Langevin dynamics, followed by a short (5 ns) equilibration process at constant pressure (1 atm) and temperature (300 K). Finally, unbiased and unrestrained system went through production in a periodic boundary condition for 500 ns in NPT by Langevin thermostat and Monte Carlo barostat conditions. Bonds involving hydrogen were constrained by the SHAKE algorithm. Cpptraj (Roe and Cheatham, 2013) was used to analyze the root mean square deviation (RMSD) and Dynamical Cross Correlation (Kamberaj and Vaart, 2009) within the ensembles comparing them to the wild type complex.

### 2.3 Structure predictions with AlphaFold

We used Alphafold Multimer (Evans et al., 2021b) to predict the structure of the complex using either sequence data or templates (containing the collagen and integrin domain far from each other). Results were analyzed in terms of the predicted local distance difference test (pLDDT) score as is standard in the field (Jumper et al., 2021). In short, the pLDDT score gives a per residue and global value to show how confident the Alpha Fold prediction results are. Results above 80 typically reflect high confidence in the prediction.

### 2.4 Thermodynamic integration (TI) calculations

TI was used to calculate the relative binding affinity ( $\Delta \Delta G_{\mathrm{bind}}^{\mathrm{mutant-WT}}$ ) between collagen and *α*2*β*1 upon mutation of certain residues in collagen. Here, we applied “One-step” transformations (Steinbrecher et al., 2011) to decrease the simulation time, in which electrostatic and van der Waals forces are varied synchronously (Shirts et al., 2003). The initial system was prepared using AMBER’s *tiMerge* to eliminate redundant bonding terms and increase calculation efficiency. We ran TI simulations with *pmemd*. The complex and mutant ligand were solvated separately in a cubic box with explicit OPC (Izadi et al., 2014) water and a 10 Å clearance. We employed ff14SB (Maier et al., 2015) for the protein parameters and general AMBER force field (He et al., 2020) for general atom and bonds parameters. Minimization, heating and equilibrium process was performed in the NVT ensemble with a Monte Carlo barostat. The TI production phase was done in the NPT ensemble (300 K and 1 atm), running for 500 ns Softcore potentials were applied to reduce issues with the integration step at the endpoints (Steinbrecher et al., 2011). Eleven independent MD simulations were performed spaced evenly between the end-points (*λ* ∈ [0, 1]). We performed six replicates for each simulated system. The average and standard deviation for ΔΔ*G*_*bind*_ were calculated from the differences amongst replicates.

### 2.5 Sampling collagen binding modes with MELD

The Modeling Employing Limited Data (MELD) approach uses H,T-REMD (Sugita and Okamoto, 1999) to sample rare events. The method changes the Hamiltonian by enforcing information that guides to different conformations that might be compatible with the end state. The caveat is that the data is framed as ambiguous and noisy—thus MELD relies on Bayesian inference to identify the best interpretation of the data compatible with the forcefield. In this process, analyzing the resulting ensemble (e.g., through clustering) identifies the states (conformations) most compatible with the information and force field.

To guide the binding process we first placed harmonic distance restraints amongst native contacts in the integrin (so it would not unfold), and also between the three collagen strands, so it would not dissociate. We then selected residues in the active site of the integrin and in those of the collagen binding motif. Based on those two lists of residues, we generated a list of twenty five possible contacts (some of which were present in the native state and some of which were not). We found that when enforcing 15 or more restraints, replica exchanges were inefficient, leading to poor sampling. At the other extreme, satisfying less than four restraints sampling was not restrictive enough to sample native-like bound conformations. We thus required that only eight restraints out of the 25 possible ones be satisfied. Satisfying different subsets of eight restraints give rise to different binding modes.

MELD simulations used the ff14SB force field (Maier et al., 2015) for side chains and ff99SB (Hornak et al., 2006) for backbone, together with the GBneck2 (Nguyen et al., 2013) implicit solvent model. The collagen fiber was placed over 30 Å away from the integrin. The temperature range was set between 300 and 500 K, with 30 replicas. Ensembles were analyzed using hierarchical clustering as implemented in CPPTRAJ (Roe and Cheatham, 2013) with an *ϵ* = 2 value, including heavy atoms at the interface of the complex in the native state.

## 3 Results

### 3.1 Local search for new interaction motifs

**FIGURE 2:**
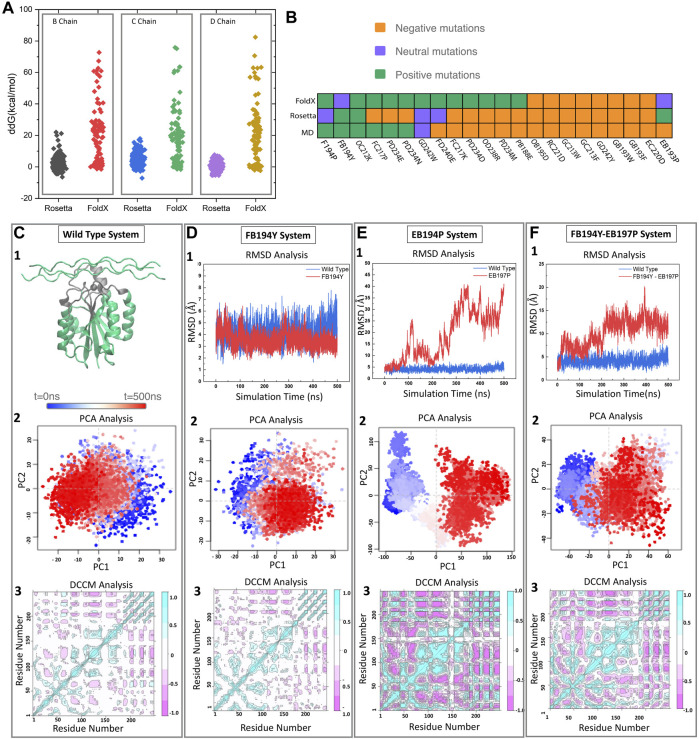
Pipeline for selecting new motifs. **(A)** FoldX and Rosetta are used to estimate relative free energy changes upon mutating each residue in the binding motif to all possible amino acids. **(B)** Predicted effect of mutations by three different methods (FoldX, Rosetta, and MD) for a set of 22 mutations. **(C)** The wild type samples a single state throughout the trajectory as identified by projecting onto the two first principal components. Reference Dynamic cross correlation matrix (DCCM) for the wild type state. **(D–F)** Examples of a stable **(D)** and unstable **(E,F)** mutations as identified from RMSD, PCA, and DCCM.

Traditional design strategies start with a known binding motif and search for single amino acid mutants that increase binding affinity (ΔΔ*G*_*bind*_). Such strategies lead to local sequence optimization, with designs similar to the original motif. Here we used FoldX (Schymkowitz et al., 2005) and Rosetta (Barlow et al., 2018) (see methods), two traditional approaches with varying computational cost and success rate. We observed that FoldX single point mutations have a wider ΔΔ*G*_*bind*_ distribution, and are generally shifted towards higher energies (see Figure 2A). While there is good agreement on the failed mutations, the more computationally demanding Rosetta is better at discriminating mutations that FoldX finds favorable.

To further assess the predicted motifs with an independent methodology, we performed MD simulations of a selected group of 15 mutants. We expect that monitoring standard structural and dynamical properties like RMSD and dynamical cross correlation functions would be enough to distinguish those mutations that remain stable in the 500 ns timescale vs. those that are unlikely to bind (see Figures 2C–F). We monitored the RMSD of the interface region, defined as heavy atom contacts to collagen in the native structure (using a 10 Å cutoff). In this timescale, the integrin oscillates around 1 Å from the initial structures, with few deviations to higher RMSD values (2.5 Å). In the presence of collagen we observe a similar behavior, where there are no deviations to larger RMSD states in the 500 ns timescale. The RMSD of the whole complex oscillates at around 4 Å. Figure 2 showcases the behavior of the wild type, neutral and negative mutation on sampling [RMSD and projection on the top two principal components using the Bio3d package (Grant et al., 2006)]. Figure 2 exemplifies a negative control mutation (which rapidly dissociates) and a neutral mutation that remains close to the starting conformation.

We find that the more computationally efficient FoldX is capable of filtering out mutations that are likely detrimental to the binding affinity. While the ones predicted to be beneficial do not always agree with MD and Rosetta results (see Figure 2B). We notice several disagreements with Rosetta and MD—this is not surprising as Rosetta has been designed to predict free energy differences while short conventional MD trajectories do not contain enough sampling to assess the free energy. We thus decided to perform thermodynamic integration calculations to further identify the agreement between Rosetta and MD-based approaches.

Thermodynamic integration increases the complexity in system setup and analysis with respect a conventional MD trajectory—but the computational costs (considering replicates needed, see methods) remains relatively small compared to other MD approaches. We selected 15 mutations and compared results using Rosetta and TI (see Supplementary Figure S1). For most residue mutations, both programs agree in sign if not in magnitude. Previous work points to systems including multiple binding modes or systems that are sensitive to local conformational changes (such as the MIDAS binding site) (Armacost et al., 2020) as problematic for TI. For example, Guest and coworkers performed free energy perturbation studies on a series of small molecule inhibitors to the *β*6 integrin with an average error of 1.5 kcal/mol with respect to the experimental results (Guest et al., 2020).

We searched for alternative binding modes by using the MELD approach, which can simulate multiple binding/unbinding events. MELD combines ambiguous/noisy information with molecular simulations through Bayesian inference and has been routinely used for predict the binding of macromolecules [protein-protein (Brini et al., 2019), protein-peptide (Morrone et al., 2017; Mondal et al., 2022), protein-DNA (Bauzá and Pérez, 2021), and protein-small molecule (Liu et al., 2020)]. We derived ambiguous information based on native contacts present in the crystal structure in such a way that different interpretations of the data is compatible with different binding modes. We expected, that the force field would be able to recognize the most native-like amongst the binding modes for those sequences that have a high affinity (clusters with high population) (Lang and Perez, 2021). Unfortunately, due to the small interface region between collagen and the integrin, the different binding modes found give rise to large deviations in binding angles between the collagen in MELD simulations with respect to the native structure (see Supplementary Figure S2). On the other hand, satisfying more information overrides the force field preferences and yields native-like binding modes regardless of the sequence. Similarly, competitive binding simulations (Morrone et al., 2017) with MELD also failed to distinguish which collagen mutations were more likely to lead to more stable complexes. Presumably, these limitations arise from the use of an implicit solvent (Nguyen et al., 2013) needed for the MELD binding simulations.

Similarly, the recent successes of the AlphaFold (AF) (Evans et al., 2021a) machine learning approach did not translate to this system. We used a local installation of AlphaFold and performed predictions in the presence/absence of structural templates. In our hands, Alphafold multimer predictions were confident about the *α*2 I-domain structure (high pLDDT scores), but failed to predict the structure of the collagen triple helix structure—and hence of the complex (see Supplementary Figure S2).

### 3.2 Recent machine learning approaches can suggest novel sequences based on the structure

Whereas we used FoldX and Rosetta to predict local changes in the sequence (single mutants), the recent protein MPNN (Dauparas et al., 2022) machine learning approach can in principle find an optimal sequence given the structure of the complex. Contrary to the other two methods, this approach does not provide a relative binding affinity. We first generated two predictions in which we allowed any residue in the motif along the tree collagen strands to change (see “Prediction 1″ and “Prediction 2” in Supplementary Figure S3). This gave rise to four different binding motifs. We next generated four more sequences by creating homo-trimer collagen strands with each of the four predicted motifs (see the latter four motifs in Supplementary Figure S3A). We assessed the viability of these motifs by running conventional MD. All sequences in which the leading strand had an E to P mutation were unstable. Whereas if this mutation occurred in other strands, the system remained stable. This is expected as the Glutamic acid coordinates with a divalent site when interacting with the integrin.

## 4 Discussion

In this work we focused on identifying collagen-like motifs that bind the I-domain of the *α*2*β*1 integrin. Despite their biological relevance and some successes (Craig et al., 2004; Murcia et al., 2008; Choi et al., 2010; Zhu et al., 2010; Wang et al., 2015; Farina et al., 2016; Fratev and Sirimulla, 2019), integrins remain challenging systems to study through molecular modeling. The collagen fiber with the *GFOGER* motif that we study was initially suggested based on docking calculations (Emsley et al., 2000), which led to the crystallization of the complex (pdb code 1dzi). Our use of local (single mutant) and global (proteinMPNN) approaches shows that current methodologies are better at discerning unfavorable mutations than at providing reliable predictions. However, consensus between different methods increases the likelihood of success. Our use of MD stability analysis showed that it can be a helpful tool to distinguish unfavorable mutations, but stable simulations are not a guarantee of favorable mutations as timescales remain limited. This becomes an issue even when using thermodynamic integration, as multiple binding modes are possible. While this is an actively developed field for small molecule binders Gill et al. (2018), it remains more challenging for flexible molecules such as collagen. For such flexible systems, we have previously found the MELD Bayesian inference approach can typically identify differences amongst different binder sequences. Due to the small interface area, our standard protocol results in binding modes where the collagen binds in the right region, but with orientations that can deviate up to 90° from their experimental binding mode. The caveat of increasing the number of restraints in MELD to solve this issue leads to the inability to distinguish motif sequence preferences.

Molecular modeling pipelines are undergoing rapid and drastic changes thanks to the eruption of machine learning approaches. The CASP event served as the perfect scenario for the first iteration of AlphaFold to show the potential of machine learning in protein structure prediction (Senior et al., 2020). Their initial approach relied on following the leading strategies in the field: determine pair-wise distance distributions between residues to impose as restraints to predict structures. Two years later, AlphaFold presented a novel strategy based on attention networks with an impressive performance in CASP (Jumper et al., 2021). Making the network available to the community and the appearance of collaborative notebooks (Mirdita et al., 2022) rapidly allowed groups to apply it to a myriad of problems: for molecular recognition (protein-protein and protein-peptide) (Humphreys et al., 2021; Tsaban et al., 2022), for predicting multiple biological states (Wayment-Steele et al., 2022), relative binding affinities (Chang and Perez, 2022), or even for designing new proteins *via* deep network hallucination (Anishchenko et al., 2021). As these networks learn from data deposited in the protein data Bank, they also implicitly learn about the position of ions or ligands in active sites. However, AF multimer was not able to predict the structures of the 1dzi complex. Recent work showed that partial retraining pf AF weights for specific targets could lead to an improved ability to correctly identify bound or unbound peptides binding to the Major Histocompatibility Complex (MHC) (Motmaen et al., 2022). This was possible thanks to a large database of peptides known to be either binders/non-binders to MHC. Such type of initiatives could soon provide accurate results for predicting complexes involving integrins, which combined with competitive binding strategies (Chang and Perez, 2022) could lead to rapid identification of functional motifs.

During the writing of this paper, several new machine learning approaches appeared in the literature which make us optimistic about the future: we highlight three that are relevant to the discussion above. The first one is RosettaFoldNA (Baek et al., 2022), which predicts the folding of RNA as well as nucleic acid-protein complexes. The approach draws on the AF principles but incorporates an additional physics-inspired term (Lennard Jones potentials taken from Rosetta) to better reproduce geometries (e.g., reduce the overlap between protein and nucleic acids). In this process, the algorithm has learned to assemble double-stranded DNA, much like we hope the collagen triple helix can be predicted. The second development is the OpenFold (Ahdritz et al., 2022) initiative—a pyTorch-based implementation trainable to reproduce AlphaFold levels of accuracy at a lower computational cost. The authors also report the OpenProteinSet used to train the model. In the last few months, the field used AF beyond what it was originally designed to do. OpenFold will now give users the possibility to retrain a tool equivalent to AF for new purposes. Finally, a recent study (Akdel et al., 2022) highlights the potentially transformative role of AF in structural biology, its accuracy matching experiments for many applications, as well as the role of potential biases, and its ability to identify features that are not typically present in databases.

## 5 Conclusion

In this work we assessed the role of different computational tools to identify novel collagen-integrin binding motifs. FoldX serves as a fast mutant screen, to filter out mutations that do not improve binding affinity. A combination of Rosetta and MD (TI) serves to further identify those mutations most likely to lead to improved binding affinities. Although we were very enthusiastic about the possibility of using AlphaFold to differentiate amongst binding motifs, we found no evidence that it could predict the native state. However, in light of recent work it seems like partial retraining of the weights against known binders/non-binders might lead to a feasible pipeline. Finally, proteinMPNN was able to correctly identify that mutations to the glutamic acid involved in binding would be deleterious only in the leading strand. Although further assessment is needed, proteinMPNN paves the way to identifying functional motifs far from the starting sequence motif.

## Data Availability

The raw data supporting the conclusion of this article will be made available by the authors upon request.
