# Supplementary material for: Assessing a computational pipeline to identify binding motifs to the α2β1 integrin
Source: Front Chem. 2023 Feb 13;11:1107400. doi: 10.3389/fchem.2023.1107400 (PMC9968975; doi:10.3389/fchem.2023.1107400)
Supplement: Supplementary file 1 [file Presentation1.pdf]

Supplementary Information for:

Assessing a computational pipeline to identify binding motifs to the α2β1 integrin

Qianchen Liu[1], Alberto Perez[1, *]

[1]Department of Chemistry and Quantum Theory Project, University of Florida,
Gainesville, Florida, USA
Correspondence*:
Corresponding Author
perez@chem.ufl.edu

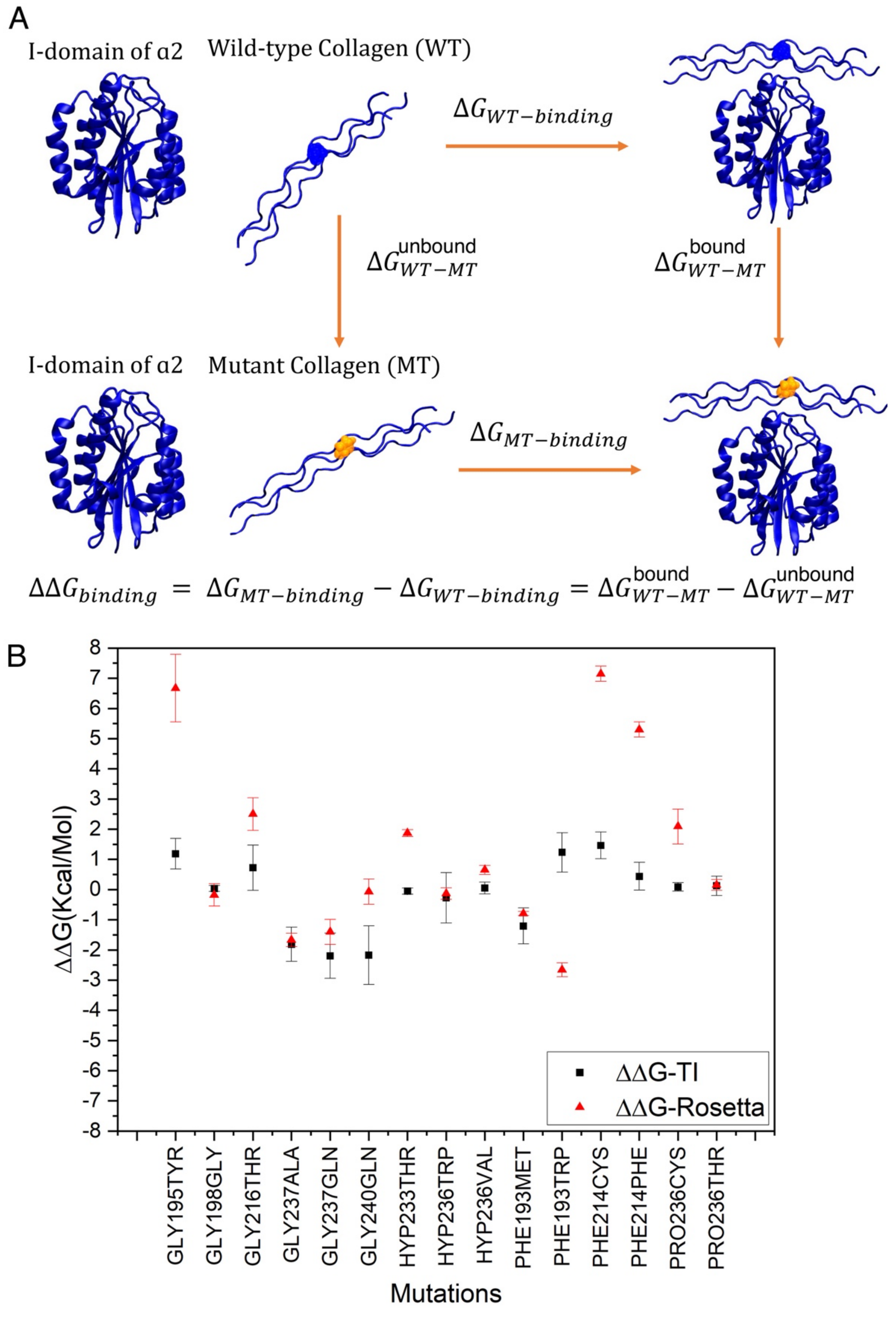

Supplementary Figure 1: Thermodynamic integration results. A. Thermodynamic cycle used for TI calculations. B. Predicted effect of mutations on binding affinity using TI and RosettaDDG.

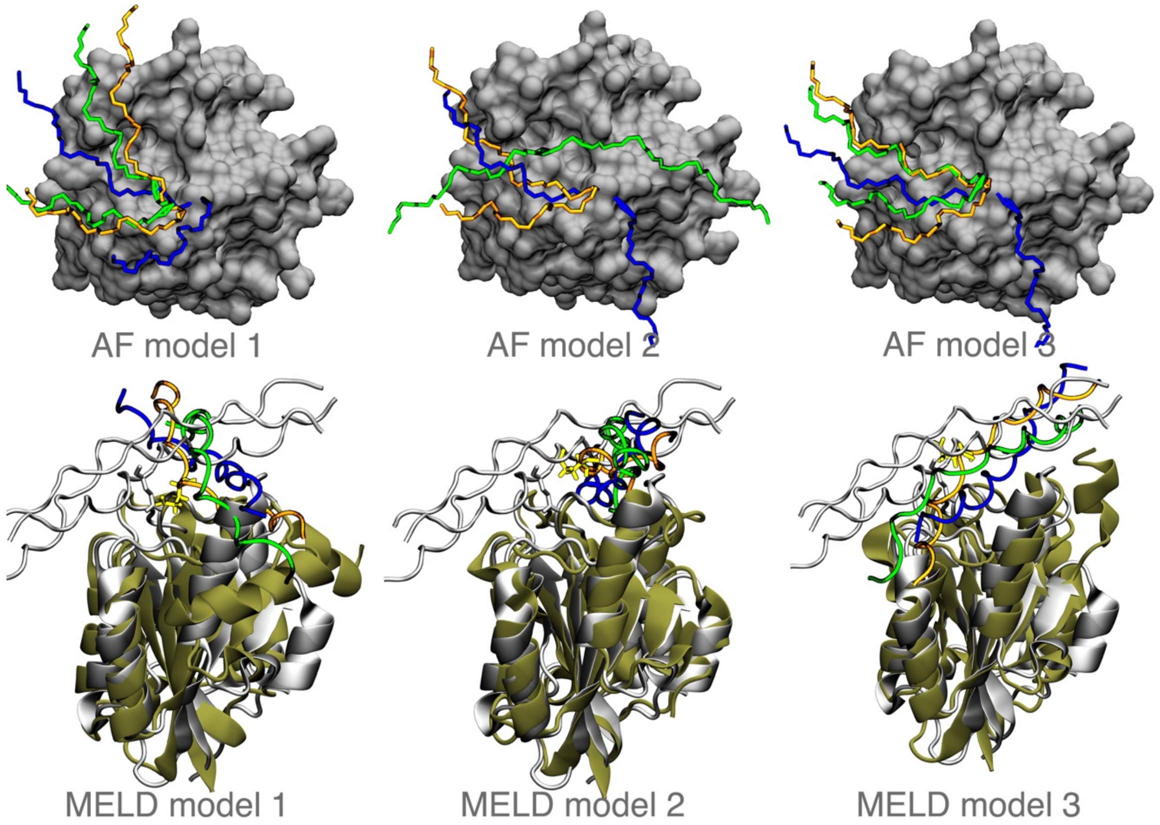

Supplementary Figure 2: Both AF and MELD fail to model the native complex. Top: three models produced by AF show significant overlap between the three collagen strands. Although the correct binding site is identified, the method tries to arrange all strands in the same binding site. Bottom: MELD predictions (tan for the protein and blue, green, orange for the three strands) show significant changes with respect to the crystal structure (grey). The glutamic acid in the leading strand is highlighted in licorice. MELD predicts the GLU interacting in the correct binding site in the first model but fails to find the right orientation for the collagen.

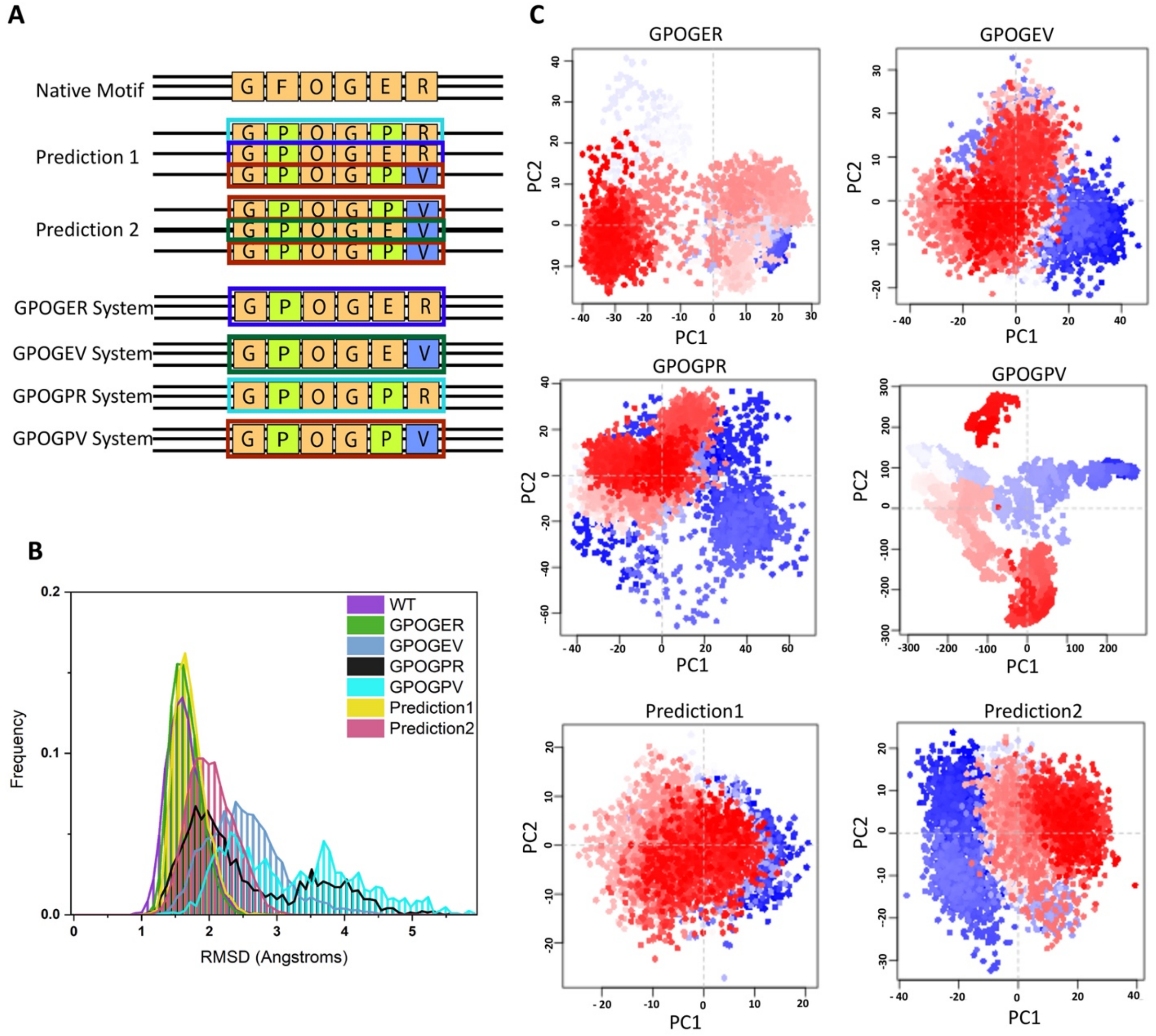

Supplementary Figure 3: Prediction results from ProteinMPNN. A. Each black line represents a strand of the collagen triple helix for either the Wild Type (native) motif or each of the predictions from proteinMPNN. For predictions 1 and 2 we allowed proteinMPNN to suggest different mutations in each strand. We took the four different motifs predicted in this way to create homo-trimers (GPOGER, GPOGEV, GPOGPR, and GPOGPV). Yellow, green, deep blue square represents conservation of the native motif (GFOGER), a phenylalanine to proline mutation, and an arginine to valine mutation respectively.

B. RMSD distribution for MD ensembles with respect the starting conformation shows that sequences which mutate E to P in the leading strand are not stable. C. PCA analysis for the six systems showing the different sampled states (each dot represents a frame, where the Blue-White-Red color code indicates starting to ending frames)
